# Changes in Urinary Aquaporin 2 and Serum Sodium After Catheterization in Elderly Patients with Syndrome of Inappropriate Antidiuretic Hormone and Urinary Retention: A Preliminary Hospital-based Study in Yangon, Myanmar

**DOI:** 10.17925/EE.2025.21.1.6

**Published:** 2025-03-17

**Authors:** Than Than Aye, Mya Thanda Sein, Phyo Thiha, Tin Myo Han, Htar Ni Aye, Yin Thu Theint, Mie Mie Pyone, Kyaw Swar Thet, Thet Htun Zaw, Aye Moh Moh Han

**Affiliations:** 1. Grand Hantha International Hospital, University of Medicine 2, Yangon, Myanmar; 2. Department of Physiology, University of Medicine 2, Yangon, Myanmar; 3. Department of Medicine, University of Medicine 2, Yangon, Myanmar; 4. Primary Care Research Unit, Myanmar GPs Society, Grand Hantha International Hospital, Yangon, Myanmar; 5. Department of Medicine (Endocrinology), Ng Teng Fong General Hospital, Singapore; 6. Grand Hantha International Hospital, Yangon, Myanmar

**Keywords:** Aquaporin 2, catheterization, elderly, hyponatraemia, retention of urine, syndrome of inappropriate antidiuretic hormone secretion (SIADH)

## Abstract

**Background:**

The syndrome of inappropriate antidiuretic hormone secretion (SIADH) is the most common electrolyte disorder among elderly patients. Chronic urinary retention has also been implicated in the development of SIADH. The mechanism by which urinary retention leads to SIADH remains unclear. Increased responsiveness of the collecting ducts to arginine vasopressin has been observed in elderly patients with urinary retention. This study aims to evaluate whether SIADH in elderly patients with urinary retention is associated with increased urinary aquaporin 2 (U-AQP2) levels and whether the insertion of an indwelling catheter with fluid restriction, without the administration of 3% saline, can lower the U-AQP2 level, leading to the resolution of SIADH.

**Method:**

This hospital-based clinical intervention study was conducted from January 2022 to January 2023. Eighteen elderly patients who met the selection criteria for euvolaemic SIADH (identified by Bartter and Schwartz criteria) associated with urinary retention, after excluding other causes, were selected. Serum sodium (Nas), serum osmolality (Osms), U-AQP2 levels, urinary osmolality (Osmu) and 24-hour urine volume on days 1 and 4 post-catheterization were assessed and compared. Clinical responses, including neurological signs and symptoms (Glasgow Coma Scale [GCS]), were also recorded.

**Results:**

All 18 cases had comorbidities and were in a range of severe hyponatraemia, defined as Nas<125 mmol/L. Nas levels significantly increased (p<0.05) on days 2 and 4 after the drainage of residual urine, with mean (± standard deviation) changes of 8.39 (± 5.7) and 15.67 (± 5.6) mmol/L, respectively, from a baseline of 110.7 mmol/L. Osms significantly increased (p<0.05) from 240.01 (± 15.68) mOsm/kg on day 1 to 272.74 (± 13.41) mOsm/kg on day 4 post-catheterization. The mean urinary aquaporin:creatinine ratio significantly decreased (p<0.05) from 3,348.01 (± 2,127.82) fmol/mg Cr on day 1 to 1,135.27 (± 1,194.42) fmol/mg Cr on day 4. The mean Osmu significantly decreased (p=0.00) from 450.67 (± 187.3) mOsm/kg on day 1 to 229.33 (± 123.56) mOsm/kg on day 4. The mean urine volume significantly increased (p<0.05) from 1,610.00 (± 530.15) mL on day 1 to 2,725.56 (± 898.29) mL on day 4. All patients showed neurological improvement, with the mean GCS increasing from 11 to 14, without complications of osmotic demyelination syndrome.

**Conclusion:**

U-AQP2 levels are elevated in elderly patients with SIADH with urinary retention. After catheterization, these levels decrease, leading to the spontaneous resolution of hyponatraemia without complications.

Hyponatraemia is primarily a disorder of water balance or distribution, characterized by serum sodium (Nas) levels less than 135 mmol/L.^1^ Hyponatraemia is the most common electrolyte disorder among elderly patients and is associated with increased mortality rates and longer hospital stays.^2–4^ The syndrome of inappropriate antidiuretic hormone secretion (SIADH) is a frequent cause of hyponatraemia in this population, posing substantial clinical challenges due to its complex aetiology and management.^5^ Elderly patients are especially vulnerable to the adverse effects of hyponatraemia, including cognitive dysfunction, falls, fractures and increased mortality rates.

SIADH is associated with various underlying conditions, such as malignancies, pulmonary disorders and central nervous system diseases. Notably, bladder outlet obstructions (BOOs) and urinary retention have also been implicated in the development of SIADH, as evidenced by several case reports. Hyponatraemia was corrected rapidly following urinary catheterization.^5–14^

Than Than Aye and Toe Lwin reported an association between hyponatraemia and urinary retention, noting that drainage of retained urine resolved the hyponatraemia without the need for salt replacement therapy.^15^ Nevertheless, SIADH associated with urinary retention has not been explicitly recognized as a cause in the existing literature. This oversight suggests that this issue remains clinically underdiagnosed.

The mechanism by which urinary retention leads to SIADH is assumed to involve increased arginine vasopressin (AVP) secretion from the pituitary. This is thought to be related to the pain caused by the stretching of the bladder wall, which can activate the sympathetic nervous system and lead to the inappropriate release of ADH. Another possible mechanism is the increased responsiveness of the collecting duct via an increased expression of aquaporin-2 (AQP2).

AQP2 in urine was first identified by Kanno et al. (1995), and the mechanism of exosome-m ediated urine excretion of AQP2 was discovered by Pisitkun, Shen and Knepper (2004).^16,17^ Urinary aquaporin 2 (U-AQP2) and plasma AVP levels are positively correlated, with the kidney excreting about 3% of its total AQP2 content per day while at rest.^16^ AQP2 trafficking is tightly controlled by the pituitary hormone AVP, which stimulates the translocation of AQP2 from storage vesicles to the apical membrane.^18^ Thus, the measurement of urinary excretion of aquaporin-2 provides a marker of collecting duct responsiveness to vasopressin in SIADH.

Ishikawa et al. found that urinary excretion of AQP2 is a more sensitive measure of AVP’s effect on renal collecting duct cells than plasma AVP levels.^19^ They also observed that increased urinary excretion of AQP2 indicates exaggerated AVP-i nduced antidiuresis in elderly hyponatraemic subjects.^19^ Enzyme-l inked immunosorbent assay (ELISA) is one of the methods for quantitation of U-AQP2, and it has an excellent correlation.^20^

The assessment of U-AQP2 levels has not been extensively studied in the context of SIADH related to urinary retention. Understanding these changes could elucidate the mechanisms underlying the resolution of hyponatraemia post-catheterization as a management strategy for affected patients. Hence, this study aims to investigate the alterations in U-AQP2 levels and the subsequent recovery of hyponatraemia in elderly patients with SIADH secondary to urinary retention, before and after bladder decompression.

By exploring these parameters, we hope to enhance the clinical recognition and treatment of this underappreciated cause of SIADH.

## Methodology

### Patients

This hospital-based clinical intervention study enrolled consecutive elderly patients with hyponatraemia due to SIADH secondary to urinary retention, admitted to Grand Hantha International Hospital (GHIH) in Yangon, Myanmar, from January 2022 to January 2023. Out of 80 elderly patients admitted to GHIH with hyponatraemia during the study period, 18 cases meeting the selection criteria were included. Participation was voluntary. Written informed consent to participate in the study was obtained from all study participants or their next of kin if the participant was unable to provide consent.

The diagnosis of SIADH was based on the Bartter and Schwartz criteria: Nas <135 mmol/L, decreased serum osmolality (Osms; <275 mOsm/kg), inappropriately concentrated urine (>100 mOsm/kg) and urine sodium concentration >20 mmol/L.^21^ Elderly patients with SIADH and urinary retention, having residual urine of more than 50 mL as detected by ultrasound or simple catheterization, were selected.

The study was approved by the Ethics Review Committee of the University of Medicine 2, Yangon, and conducted in accordance with the principles outlined in the Helsinki Declaration of 1964 and its later amendments. The study protocols were approved by the Republic of Union of Myanmar Ministry of Health, Yangon, Myanmar, Letter No. 48/ETC-1 (1-2021) dated 14 October 2021.

Exclusion criteria of the study included:

- hyponatraemia or SIADH from other causes such as carcinoma, hypothyroidism and hypo-adrenalism;
- history of corticosteroid usage, antipsychotic medications and diuretic usage;
- advanced chronic kidney disease (estimated glomerular filtration rate less than 45 mL/minute/1.73 m^2^);
- marked hyperproteinaemia, hyperlipidaemia or hyperglycaemia;
- patients with hypovolaemia, dehydration, hypervolaemia due to congestive heart failure and cirrhosis of the liver
- acute stroke;
- severe neurological symptoms (Glasgow Coma Scale [GCS] less than 10/15) and seizures; and
- those who need admission to intensive care.

### Management of hyponatraemia cases after hospitalization and data collection

The selected cases were managed with vigilant and close monitoring of their vital signs, consciousness levels assessed by the GCS and fluid intake–output recording. Fluid intake was restricted to 1 L/day. Indwelling catheterization was performed under strict aseptic protocols for continuous urine drainage. Conventional management of hyponatraemia, such as 3% saline administration, was withheld.

Termination from the study and a switch to the standard management protocol (e.g. 3% saline administration) were implemented in cases of clinical deterioration or at the patient’s request. To prevent osmotic demyelination syndrome, careful monitoring was done to ensure that the correction does not exceed 8–10 mmol/L/day. Comorbidities, including urinary tract infections and glycaemic control, were managed as needed.

The following parameters were assessed and observed during the study period: clinical parameters, including GCS, 24-hour urine output, Nas, Osms and urinary osmolality (Osmu), were recorded from days 1 to 4. Samples for U-AQP2 levels were collected on days 1 and 4 for further analysis. All the laboratory tests were conducted at GHIH, except the U-AQP2 assessments, which were performed at the ‘Common Research Laboratory’ at the University of Medicine 2 by the enzyme-l inked immunosorbent assay (ELISA) method.^22^ A 5 mL urine sample was centrifuged at 2,000–3,000 rpm for 20 minutes, and the supernatant was stored at -20 °C for AQP2 analysis by ELISA. Samples and reagents were prepared, added to wells and incubated for 1 hour at 37 °C. After washing the plate five times, substrate solutions A and B were added and incubated for 10 minutes at 37 °C. The stop solution was added, and the optical density value was read within 10 minutes. U-AQP2 was adjusted for urinary creatinine (UCr) by dividing U-AQP2 by UCr. U-AQP2 excretion was expressed as fmol U-AQP2/mg Cr.

### Data collection

Socio-demographic and clinical background data, along with clinical signs and symptoms, were collected using a pretested proforma. All participants and their families were thoroughly informed about the data collection procedure in detail. The first instance was for determining the eligibility of participants in this study by investigating urine sodium, Nas, Osms, Osmu and residual urine. On day 4 following catheterization, the same data collection procedure was repeated.

### Statistical data analysis

Frequency (%) was applied for the analysis of nominal and categorical data: gender, age groups, comorbidity status and presenting symptoms. Summary descriptive analysis, including range, means and standard deviations (SDs), was used for continuous numerical data. Trends and changes in Nas, U-AQP2, Osmu, Osms, urinary aquaporin:creatinine ratio (U-AQP2/Cr) and urine volume were analysed using the paired samples *t*-test. A two-tailed test with a significance level of 0.05 was set for inferential data analysis.

## Results

A total of 18 elderly patients who met the inclusion and exclusion criteria were recruited from 80 elderly patients admitted to GHIH, Yangon, Myanmar from January 2022 to January 2023. Out of these 18 patients, 83.3% (15/18) were female.

All patients had comorbidities. The most common comorbidity was hypertension (94.4%), followed by diabetes (83.3%), neurogenic bladder (50%) and urethral stricture (44.4%). Among the 18 patients, 83.3% (15/18) presented with hyponatraemia symptoms, including nausea and vomiting (83.3%), loss of energy and fatigue (66.7%) and headache (33.3%). Urinary signs and symptoms included frequency and nocturia (83.3%), urgency and incontinence (44.4%) and difficulty in micturition (38.9%). GCS scores of patients on admission ranged from 10 to 12. Background demographic data, comorbidities, clinical features, GCS score and residual urine of the patients on admission are presented in *Table 1*.

**Table 1:** Background demographic data, comorbidities, clinical presentation and Glasgow Coma Scale score

Variables	n	%
**Age**		
Minimum age	65 years	
Maximum age	91 years	
**Gender**		
Male	3	16.7
Female	15	83.3
**Comorbidity**		
Yes	18	100
No	0	0
**Comorbidities**		
Hypertension (+)	17	94.4
Diabetes (+)	15	83.3
Urethral stricture/obstructive uropathy	8	44.4
Neurogenic bladder		
Spine problems	9	50
Fractures/PID/spondylosis	3	16.7
Others		
Aasthma, pulmonary fibrosis/IHD/ UTI/ HBV	13	72.2
**Presenting clinical features**		
**Hyponatraemia symptoms**		
Nausea and vomiting (+)	15	83.3
Loss of energy and fatigue (+)	12	66.7
Headache (+)	6	33.3
**Urinary signs and symptoms**		
Urgency and incontinence	8	44.4
Difficulty in micturition (+)	7	38.9
Frequency and nocturia (+)	15	83.3
**GCS on admission (day 1)**		
GCS 10	7	38.9
GCS 11	4	22.2
GCS 12	7	38.9
**GCS on 4 days after urinary catherization (day 4)**		
GCS 13	5	27.8
GCS 14	2	11.1
GCS 15	11	61.1
**Amount of residual urine on catheter insertion**		
Minimum	100 cc	
Maximum	1,500 cc	
Mean ± SD	310 ± 325.9 cc	

*Background demographic data, comorbidities, clinical presentation and GCS score of the patients (n=18).*

*GCS = Glasgow Coma Scale; HBV = hepatitis B virus; IHD = ischaemic heart disease; PID = prolapsed intervertebral disc; SD = standard deviation; UTI = urinary tract infection.*

**Figure 1:**
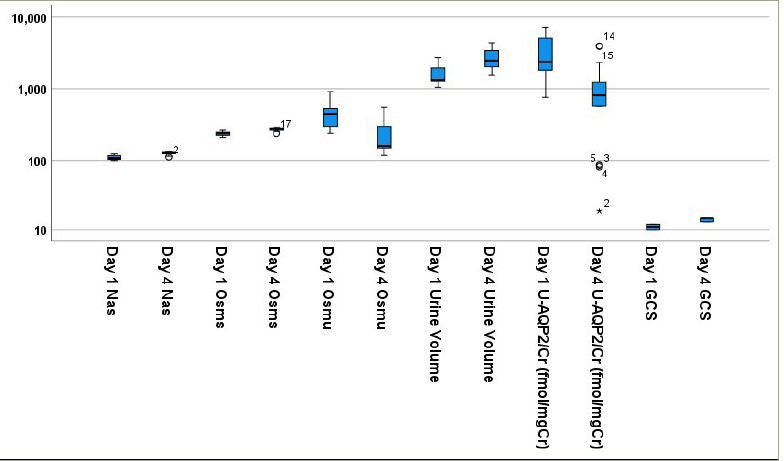
Biochemical parameter changes on admission and 4 days after urinary catheterization

In the study, Nas levels of the patients were measured on admission (day 1), day 2 and day 4 after drainage of residual urine. The mean (± SD) Nas on day 1 was 110.7 mmol/L (± 7.9), ranging from 100 to 123 mmol/L. All selected cases were found to be less than 125 mmol/L (severe hyponatraemia). After catheterization, the mean (± SD) Nas levels of days 2 and 4 were 119.11 (± 7.5) and 126 (± 6.09), respectively. Mean (± SD) changes in Nas levels from day 1 to day 2 and day 4 were -8.39 (± 5.7) and -15.67 (± 5.6), respectively. Paired samples comparison of Nas level changes in 18 patients on day 1 with that of days 2 and 4 after drainage of residual urine were significantly increased (p<0.05), as shown in *Figure 1*.

U-AQP2/Cr of 18 patients was also measured on admission (day 1) and on day 4 after drainage of residual urine. The mean (± SD) U-AQP2/Cr on day 1 was 3,348.01 (± 2,127.82) fmol/mg Cr, ranging from 761.45 to 7,344.83 fmol/mg Cr. After catheterization, the mean of day 4 was 1,135.27 (± 1,194.42), ranging from 18.95 to 4,047.19 fmol/mg Cr, respectively. The mean (± SD) change in U-AQP2/Cr from days 1 to 4 was 2,212.74 (± 1,644.49) fmol/mg Cr. A paired-samples comparison of U-AQP2/Cr changes in 18 patients on day 1 with that of day 4 after drainage of residual urine were significantly decreased (p<0.05), as shown in *Figure 1*.

*Biochemical parameter changes of 18 patients on admission (day 1) and 4 days after urinary catheterization (day 4). GCS = Glasgow Coma Scale; Nas = serum sodium; Osms = serum osmolality; U-AQP2/Cr = aquaporin:creatinine ratio.*

Osmu of 18 patients under the study was also measured on admission (day 1) and on day 4 after drainage of residual urine. The mean (± SD) Osmu on day 1 was 450.67 (± 187.3) mOsm/kg, ranging from 240 to 900 mOsm/kg. After catheterization, the mean (± SD) Osmu of day 4 was 229.33 (± 123.56) mOsm/kg, ranging from 118 to 550 mOsm/kg, respectively. The mean (± SD) change in Osmu from days 1 to 4 was 221.33 (± 132.49) mOsm/kg. A paired-samples comparison of Osmu changes in 18 patients on day 1 with that of day 4 after drainage of residual urine was significantly decreased (p<0.05), as shown in *Figure 1*.

Osms of 18 patients under the study was also measured on admission (day 1) and on day 4 after drainage of residual urine. The mean (± SD) Osms on day 1 was 240.01 (± 15.68) mOsm/kg, ranging from 210.68 to 264.65 mOsm/kg. After catheterization, the mean (± SD) Osms on day 4 was 272.74 (± 13.41) mOsm/kg, ranging from 239 to 294 mOsm/kg, respectively. The mean (± SD) change in Osms from days 1 to 4 was -32.72 (± 12.25) mOsm/kg. A paired-samples comparison of Osms changes in 18 patients on day 1 with that of day 4 after drainage of residual urine was significantly increased (p<0.05), as shown in *Figure 1*.

Urine volume of 18 patients under the study was also measured on admission (day 1) and on day 4 after drainage of residual urine. The mean (± SD) urine volume on day 1 was 1,610.00 (± 530.15) mL, ranging from 1,050 to 2,750 mL. After catheterization, the mean (± SD) urine volume of day 4 was 2,725.56 (± 898.29), ranging from 1,550 to 4,500 mL, respectively. Mean (± SD) changes in urine volume from days 1 to 4 were -1,115.6 (± 615.24) mL. A paired-samples comparison of urine volume changes in 18 patients on day 1 with that of day 4 after drainage of residual urine was significantly increased (p<0.05), as shown in *Table 2*.

## Discussion

This study was conducted from January 2022 to January 2023. A total of 18 patients meeting the selection criteria for euvolaemic SIADH associated with urinary retention were managed solely with an indwelling catheter and fluid restriction, without saline infusion. Clinical responses, along with Nas, Osms, U-AQP2, urine volume and Osmu, were analysed on days 1 and 4 post-catheterization.

Females constituted the majority in this study (83.3 versus 16.7%). Elderly females are more susceptible to developing hyponatraemia due to oestrogen-i nduced stimulation of AVP secretion. Additionally, they may exhibit greater sensitivity to ADH, which is associated with an increased expression of renal vasopressin receptors.^23–25^

Khine Oo Zin’s study on gender differences in U-AQP2 excretion found that women had significantly higher 24-hour U-AQP2 levels (1,399.7 [609.4–2,246.1] pmol) than men (804.9 [436.4–1,239.1] pmol) (p<0.05). After a salt load, the percentage increase in U-AQP2 was also greater in women (563% [121.5–831.8%]) compared with men (26% [-37.0 to 172.8%]) (p<0.05).^26^

Among the associated comorbidities, chronic diabetes (83.3%), neurogenic bladder (50%) and postmenopausal urethral strictures (44.4%) in females were the important causes of urinary retention in this study. The high prevalence of diabetes mellitus in this study is likely influenced by the main researcher’s specialization as an endocrinologist. The presenting features of SIADH included neurological symptoms, and only patients with a GCS score as low as 10/15 were included in this study. Headache was present in one-third of patients (33.3%). Nausea and repeated vomiting were common symptoms (83.3%), often mimicking gastrointestinal diseases. Fatigue, also common in our patients (66.7%), could contribute to falls and fractures in the elderly. In elderly patients, these symptoms should be considered indicators of hyponatraemia, prompting early and accurate diagnosis. Regarding urological symptoms, urinary frequency and nocturia were the most common, occurring in 83.3% of cases. Urgency and overflow incontinence were also common complaints, reported by 44.4% of patients. These bladder symptoms are often ignored and left unattended, assumed to be due to poor sphincter control associated with old age.

**Table 2:** Biochemical parameter changes on admission and after urinary catheterization

Biochemical parameters	On admission (day 1) (before catheterization) mean (± SD)	Day 4 (after catheterization) mean (± SD)	Mean difference (± SD)	p-value
Nas	110.72 (± 7.91)	126.39 (± 6.09)	-15.667 (± 5.63)	<0.05
Osms	240.01 (± 15.68)	272.73 (± 13.41)	-32.72 (± 12/25)	<0.05
U-AQP2/Cr	3,361.79 (± 2,113.037)	1,135.27 (± 1,194.42)	2,226.53 (± 1,627.8)	<0.05
Osmu	450.67 (± 187.29)	229.33 (± 123.559)	221.333 (± 132.5)	<0.05
Urine volume	1,610 (± 530.15)	2,725.56 (± 898.29)	-1,115.56 (± 615.2)	<0.05

*Biochemical parameter changes in 18 patients on admission and 4 days after urinary catheterization.*

*Nas = serum sodium; Osms = serum osmolality; Osmu = urine osmolality; SD = standard deviation; U-AQP2/Cr = aquaporin:creatinine ratio.*

In our patients, the mean Nas level on day 1 of admission before catheterization was 110.72 (± 7.9) mmol/L, ranging from 100 to 123 mmol/L. The mean (± SD) Osms on day 1 was 240.01 (± 15.68) mOsm/kg, ranging from 210.68 to 264.65 mOsm/kg. Notably, although all cases fell within the range of severe hyponatraemia, the severity of neurological symptoms in the selected cases, as indicated by the GCS, was not severe, with scores of 10/15 and above. This is likely due to neurological adaptation to chronic hyponatraemia, which reduces the symptoms of cerebral oedema. This discrepancy in the severity of neurological symptoms and degree of hyponatraemia should alert clinicians to the possibility of chronic hyponatraemia, emphasizing the importance of preventing osmotic demyelination by avoiding the rapid correction of the condition.

After indwelling catheterization and fluid restriction, Nas levels increased in all participants. The mean sodium change was 8.39 (± 5.7) mmol/L within 1 day and 15.67 (± 5.6) mmol/L within 4 days from a baseline of 110.72 (± 7.9) mmol/L. Therefore, there was no rapid correction of hyponatraemia, as the increase did not exceed 10 mmol/L/day, remaining within the safety range to avoid the complication of osmotic demyelination. Sodium levels were self-corrected soon after indwelling catheterization. Hence, it is important not to prescribe salt replacement, particularly 3% saline infusion, concurrently with indwelling catheterization to avoid the risk of overcorrection in such patients.

The mean U-APQ2/Cr on day 1 was 3,348.01 (± 2,127.82) fmol/mg Cr, ranging from 761.45 to 7,344.83 fmol/mg Cr before catheterization. After catheterization, the U-AQP2 levels and the urine osmolality were significantly reduced on day 4 post-catheterization in all patients. Conversely, 24-hour urine output increased significantly in all cases. These findings, consistent with the post-obstructive polyuria following the drainage of urinary obstruction, can be explained by a reduction in AQP2 or AVP activity.^27^

Currently, there are no established cutoff levels for U-AQP2 that can definitively distinguish between individuals with normal water balance and those with SIADH. One study reported a mean AQP2 of 176.3 fmol/mg Cr in healthy individuals and mean levels of 685.0 fmol/mg Cr in individuals with SIADH.^28^ Another study found levels averaging 569.9 ± 162.7 fmol/mg Cr in individuals with SIADH.^17^ Several studies have examined U-AQP2 excretion in patients with SIADH due to various factors. Collectively, these studies indicate that U-AQP2 excretion is elevated in SIADH, regardless of the underlying cause, reflecting the inappropriate secretion or action of AVP.^17^

Compared with other studies, the levels of U-AQP2 in our study of urinary retention-related SIADH were significantly higher, measuring 3,348.01 (± 2,127.82) fmol/mg Cr. This raises the question of whether the mechanisms of SIADH related to urinary retention are possibly more closely associated with U-AQP2 expression originating from the urinary tract, in addition to AVP secretion from the pituitary.

Current research indicates that bladder distension can influence aquaporin expression. A study on rat bladders demonstrated that AQP2 expression was significantly higher in bladders distended for 3 hours compared with empty bladders. Furthermore, treatment with AQP2 siRNA suppressed this increased expression, suggesting that bladder stretching can upregulate AQP2 levels.^29^ However, there is limited evidence directly linking bladder stretching to increased aquaporin levels in humans. While human urothelium has been shown to express various aquaporins, including AQP3, AQP4, AQP7 and AQP9, the specific effects of bladder stretching on these aquaporin levels have not been extensively studied.^30^ Although this study did not show evidence of AQP2 expression in the human bladder, it demonstrated that AQPs are expressed in the human urothelium, suggesting a potential role in transurothelial water and solute transport. This serves as a platform for further research to provide clarification.

Studies specifically focusing on AQP2 measurements in BOO or urinary retention associated with SIADH are limited.^30^ According to the findings in this study, measurements of U-AQP2 may serve as a hallmark of SIADH and help monitor fluid imbalance in SIADH associated with urinary retention.

The exact pathophysiological mechanism remains unclear – whether this is due to a neurological mechanism where bladder distension stimulates stress-i nduced ADH release or there are local changes in AVP responsiveness caused by increased AQP2 expression in the collecting ducts. This requires further evaluation.

Our study has some limitations, particularly due to the small sample size, which was constrained by various factors. Further research, including comparative studies with and without urinary catheterization, is necessary to validate the effectiveness of this management approach in larger cohorts.

## Conclusion

In this study, elevated U-AQP2 levels indicating SIADH occur in elderly with residual urine, either due to functional or mechanical causes. After indwelling catheterization along with fluid restriction, there was recovery of hyponatraemia within a safe range of rise, i.e. <10 mmol/L/day, with a reduction in U-AQP2 and Osmu. All 18 cases recovered clinically from SIADH in due course following catheterization without complications of osmotic demyelination syndrome. Although this study does not provide a mechanistic explanation for urinary retention-related SIADH, the findings confirm urinary retention as an aetiology of SIADH. This information will help clinicians consider relieving urinary retention as an effective management strategy for hyponatraemia due to SIADH in such cases.
